# Investigating the Mechanism of Rare-Earth Ion Incorporation into Glass–Ceramic Crystal Phases through Er^3+^ Ion Probe Characteristics

**DOI:** 10.3390/nano14181479

**Published:** 2024-09-11

**Authors:** Zhixin Chen, Wenzhe Cui, Sijun Ren, Ju Yang, Jiayu Tian, Haitao Xia, Jiajing Shen, Guozhong Ren

**Affiliations:** 1 School of Physics, Changchun Normal University, Changchun 130032, China; qx202200244@stu.ccsfu.edu.cn (Z.C.);; 2 Faculty of Medicine, Dalian University of Technology, Dalian 116024, China

**Keywords:** glass–ceramics, Er^3+^ ions, Judd–Ofelt theory, crystal phase, probe

## Abstract

Exploring the intrinsic mechanisms of rare-earth ions entering the crystal phase has great significance for finely tuning the luminescent properties of glass–ceramics. Using Er^3+^ ions as a probe, X-ray diffraction was employed to precisely measure the crystallinity of SiO_2_-PbF_2_-Er_2_O_3_ glass–ceramics synthesized under various heat treatment conditions, confirming the occurrence of a rapid crystallization process. Additionally, by combining Judd–Ofelt theory with comprehensive analyses of absorption and fluorescence spectra, we calculated the relative proportions of Er^3+^ ions present in the crystal phase. We found that the crystallization process in the glass–ceramics and the incorporation of Er^3+^ ions into the crystal phase did not occur synchronously. This discovery provides new theoretical foundations and practical guidance for understanding the mechanism of rare-earth ion incorporation into crystal phases, which is significant for the development of functional materials with specific luminescent properties.

## 1. Introduction

Rare-earth-doped glass–ceramics have garnered significant attention due to their superior properties, including high strength, excellent oxidation resistance, good optical transmittance, adjustable thermal expansion coefficients, and low electrical conductivity. Their unique *f*-*f* transition spectroscopic characteristics, especially, spanning the visible to the near mid-infrared range [1], offer extensive application prospects in the defense, aerospace, electronics, power generation, and biomedical fields [2,3,4,5]. In glass–ceramics, rare-earth ions primarily reside within the crystal phase, and their concentration directly impacts the luminescent performance of the material. Therefore, understanding the specific mechanisms by which rare-earth ions enter the crystal phase is important.

Existing studies indicate that the doping concentration of rare-earth ions significantly influences the formation of the crystal phase, which in turn affects the luminescence intensity [6]. During the transformation from glass to glass–ceramics, the conditions of heat treatment are critical; excessively high temperatures can inhibit the formation of the crystal phase [7]. During this heat treatment, rare-earth ions migrate into the crystal phase. However, research on whether this migration process occurs synchronously with the crystallization of the glass is still limited.

The luminescence characteristics of rare-earth ions reveal information about the crystal field around them and highlight their unique value as probes for studying their environment. In recent years, the application of rare-earth ions as probes has increased, particularly with Eu^3+^ ions, where substantial progress has been made [8,9,10]. Similarly, Er^3+^ ions, which have similar chemical properties with Eu^3+^, exhibit significant emissions in the near-infrared region and show remarkable luminescence in green (540 nm and 520 nm) and red (650 nm) wavelengths, applicable for upconversion luminescence in various matrices such as glass, glass–ceramics, single crystals, and nanocrystals [11,12]. The unique energy level structure of Er^3+^ ions allows them to achieve higher excited states through excited state absorption, cross relaxation, and energy transfer processes, thereby enhancing their potential as efficient upconversion luminescent materials and ion probes.

The phonon energy of the host matrix directly impacts the non-radiative relaxation rate of the excited states of rare-earth ions, influencing the efficiency of upconversion luminescence [13]. Selecting a matrix with lower phonon energy, such as fluorides, can help enhance the intensity of upconversion luminescence. By thermally treating fluoride precursor glasses, fluoro-oxide glass–ceramics composed of glass and crystal phases can be obtained [14,15,16]. In these fluoro-oxide glass–ceramics, fluoride crystals are uniformly distributed within the residual oxide glass matrix [17], combining the excellent optical properties of fluoride materials with the outstanding thermal stability and mechanical properties of oxide materials [18,19]. Fluoro-oxide glass–ceramics, containing low-phonon-energy fluoride crystals, can accommodate more rare-earth ions and provide superior optical performance [20]. During heat treatment, fluoride crystals nucleate and grow, while rare-earth ions in the glass preferentially migrate into these fluoride nanocrystals [21,22,23,24].

This study uses Er^3+^ as a probe to explore the mechanism by which rare-earth ions enter the crystal phase. Er^3+^-doped SiO_2_-PbF_2_ glass–ceramics were prepared via a high-temperature solid-state synthesis method. X-ray diffraction (XRD), absorption spectra, fluorescence spectra, and Judd–Ofelt theory analysis were employed to investigate the effects of heat treatment duration on the entry of Er^3+^ ions into the glass-ceramic. By com-paring the degree of crystallinity with the content of rare-earth ions in the crystal phase, the study examines the asynchrony between the glass crystallization process and the process of dopant ions entering the crystal phase. This research provides a theoretical basis for optimizing heat treatment processes to improve the luminescence efficiency of rare-earth-doped glass–ceramics and expands the scope of applications for rare-earth ions as probes.

## 2. Materials and Methods

### 2.1. Materials and Sample Preparation

Glass and glass–ceramic samples were prepared using the high-temperature solid-state method. Raw materials were accurately weighed to a total of 15 g with a molar composition of 45SiO_2_-55PbF_2_-1Er_2_O_3_. SiO_2_ and PbF_2_ were of analytical grade purity, while Er_2_O_3_ was 4N high purity. The weighed raw materials were ground in an agate mortar for one hour to ensure a uniform distribution of components. Subsequently, the mixed raw materials were placed in a crucible and then heated in a silicon carbide rod furnace at 930 °C for 15 min. Upon the completion of the melting process, the melt was immediately poured onto a preheated copper plate and covered with another copper plate to achieve rapid cooling and shaping, resulting in the glass sample.

A portion of the glass sample was placed in a crucible containing alumina powder and subjected to heat treatment in a silicon carbide rod furnace. After heat treatment, the samples were naturally cooled to room temperature. Both heat-treated samples and the original glass samples were collected for subsequent testing.

### 2.2. Characterization Techniques

The samples were analyzed by a 2500PC X-ray diffraction analyzer (Rigaku, Tokyo, Japan) with a step scanning speed of 0.25–1 s, a step length of 0.1, and a step angle of 2θ. The scanning range was 20°–60°. The glass transition temperature and crystallization temperature of the sample were obtained by using differential scanning calorimetry (SDT-2960: TA Instruments, New Castle, DE, USA). The measurements were carried out under a nitrogen atmosphere with a heating rate of 10 °C/min. The UV–visible absorption spectra of the samples were measured by a UV–visible photometer model UV-mini-1280 (Shimadzu, Tokyo, Japan) with a detection range of 200 nm–1200 nm. The excitation and emission spectra of the sample were obtained using a fluorescence spectrophotometer equipped with a 150 W xenon lamp (FL-4600: Hitachi Instrument, Tokyo, Japan). The excitation source for the spectrophotometer was a 980 nm semiconductor laser (IR-980/1–5000 mW: Raish Laser Optoelectronic Technology, Changchun, China).

## 3. Results

### 3.1. Characterization of the Degree of Crystallinity by XRD

**Figure 1:**
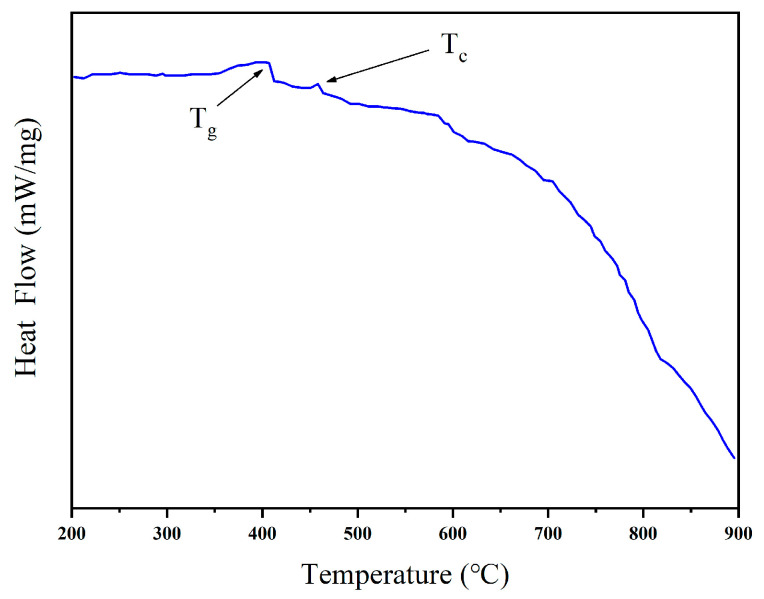
DSC pattern of Er^3+^-doped glass sample.

Figure 1 shows the differential scanning calorimetry (DSC) curve of Er^3+^-doped SiO_2_-PbF_2_ glass samples. Two critical characteristic temperatures are identified: the glass transition temperature (T_g_) and the crystallization temperature (T_c_), which are 390 °C and 430 °C. The glass transition temperature (T_g_) marks the transformation of amorphous materials from a hard and brittle glass state to a more flexible, high-elastic state, typically represented by a distinct inflection point or step on the DSC curve. The crystallization temperature (T_c_) corresponds to the temperature at which amorphous materials begin to crystallize and form an ordered structure, characterized by a significant exothermic peak on the DSC plot [25]. During the transformation of glass samples into glass–ceramics, the heat treatment temperature and time are critical for controlling the nucleation and growth of crystals. For example, crystal nuclei start forming at a heat treatment temperature of 460 °C, followed by gradual crystal growth with increased heat treatment time.

The crystallization temperatures detected by DSC were used to heat treat the glass at 460 °C for 0.5 h, 1 h, 1.5 h, 2 h, 2.5 h and 3 h, respectively, to observe the entry of Er^3+^ into the glass–ceramics as the annealing time increased [26].

X-ray diffraction (XRD) is a powerful analytical technique that provides detailed information about the crystal structure of materials, including specific details about the samples being studied. In this work, XRD was utilized to identify the crystal phases that form in annealed glass.

**Figure 2:**
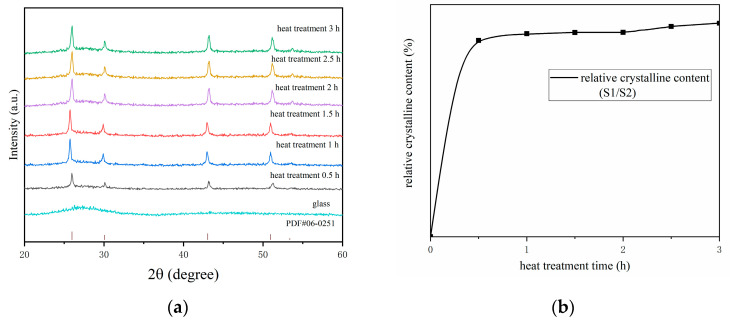
(**a**) X-ray diffraction patterns of glass and glass–ceramics; (**b**) trend curve of crystallinity over time.

Figure 2a shows the X-ray diffraction (XRD) patterns of the glass before heat treatment and of the glass–ceramics after different durations of heat treatment. The original glass sample exhibits an XRD pattern without prominent sharp peaks across all 2θ values, indicating its amorphous nature and the absence of crystal phases. After heat treatment at 460 °C for varying periods of 0.5 h, 1 h, 1.5 h, 2 h, 2.5 h, and 3 h, characteristic peaks indicative of crystal phase presence begin to appear in the XRD patterns, signaling the transformation of the glass samples into glass–ceramics. Four distinct diffraction peaks emerge, aligning closely with the peak positions in the standard card (PbF_2_, ICDD PDF#06-0251), confirming PbF_2_ as the crystal phase in the glass–ceramics. With extended heat treatment times, the positions of these four characteristic peaks remain stable, and no new diffraction peaks appear. The intensity of these characteristic peaks increases, suggesting an increase in the number of crystals. As shown in Figure 2a, the intensity of the diffraction peaks gradually increases with prolonged heat treatment time, indicating a relatively slow growth rate of PbF_2_ crystal content.

The X-ray diffraction (XRD) patterns of the samples can be used to calculate the area of each diffraction peak. As shown in Table 1, by comparing the area S1 of the crystal phase diffraction peaks with the area S2 of the amorphous phase background diffraction, one can determine the relative crystallinity (S1/S2) of the samples subjected to different heat treatment durations. The results indicate that within the first 0.5 h, the relative crystal phase content rapidly increases to 29.6%. Over the subsequent period from 0.5 h to 3 h, the content further rises to 32.2%. This suggests that the rate of increase in the relative crystal phase content is most significant during the initial 0.5 h period.

As shown in Figure 2b, the ratio of the area of the crystal phase diffraction peaks to the area of the amorphous phase background gradually increases after heat treatment at 460 °C for periods of 0.5 h, 1 h, 1.5 h, 2 h, 2.5 h, and 3 h. This indicates that the content of PbF_2_ crystals within the samples increases with prolonged heat treatment time, leading to an overall increase in crystallinity. However, this increase is very small, suggesting that the majority of the crystallization process is completed after 0.5 h of heat treatment. This finding confirms that the crystallization process in the glass–ceramics is rapid [27].

### 3.2. Calculation of J-O Parameters from Absorption Spectra

Judd-Ofelt (J-O) theory is a highly standardized and reliable analytical tool used to examine the spectral characteristics of rare-earth-doped materials. Developed independently by B.R. Judd and G.S. Ofelt in the 20th century, it has found widespread application in the qualitative analysis of rare-earth-ion-doped glasses and other materials. The J-O theory is particularly useful in characterizing radiative transitions in both doped solids and doped aqueous solutions. Its fundamental principle involves utilizing transitions between different energy levels of rare-earth ions to compute radiative parameters such as oscillator strengths, transition probabilities, fluorescence branching ratios, and radiative lifetimes from absorption spectra, within a certain degree of accuracy.

**Figure 3:**
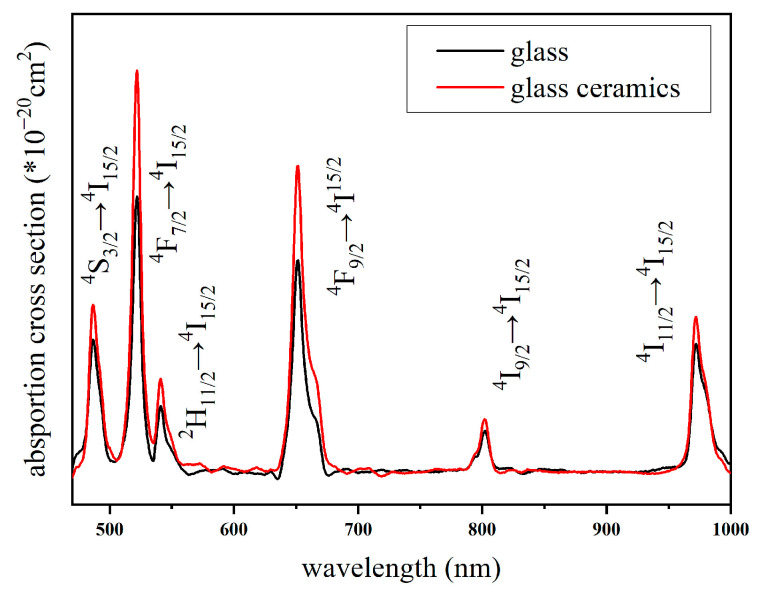
Absorption spectra of glass and glass–ceramics after treatment.

Figure 3 shows the absorption spectra of the glass and glass–ceramics over the wavelength range of 480 nm to 1000 nm, displaying six peaks corresponding to the transitions ^4^S_3/2_ → ^4^I_15/2_, ^4^F_7/2_ → ^4^I_15/2_, ^2^H_11/2_ → ^4^I_15/2_, ^4^F_9/2_ → ^4^I_15/2_, ^4^I_9/2_ → ^4^I_15/2_, ^4^I_11/2_ → ^4^I_15/2_. The corresponding peak wavelengths are 486 nm, 520 nm, 545 nm, 652 nm, 807 nm, and 972 nm, respectively. In the amorphous glass, the Er^3+^ ions are distributed within the amorphous structure, characterized by irregular arrangements of constituent ions. In glass–ceramics, due to the partial incorporation of Er^3+^ ions into the crystal phase, there is a change in the intensity of the peaks in the absorption spectra compared to those in the glass. This alteration reflects the different local environments experienced by the Er^3+^ ions in the amorphous versus the crystal regions, which in turn affects their electronic transitions and thus the observed spectral features.

According to the J-O theoretical model, the experimental oscillator strength can be calculated by the following equation:

(1) $$f_{exp}=\frac{{mc}^{2}}{N\pi e^{2}\lambda^{2}}\int \alpha (\lambda )d\lambda$$

After further derivation, it can be concluded that:

(2) $$\int \alpha (\lambda )d\lambda =\int \frac{\log_{10}\left(I_{0}(\lambda )/I(\lambda )\right)}{d\log_{10}e}d\lambda =\frac{1}{0.43d}\int OD(\lambda )d\lambda$$

where *m* = 9.1 × 10^−28^ g is the electron mass, *e* = 1.602 × 10^−19^ C is the electron charge, *c* = 2.998 × 10^8^ m/s is the speed of light, *d* is the thickness of the sample, *I*_0_ and *I* are the incident and effluent light intensities, *α*(*λ*) is the absorption coefficient, *OD*(*λ*) is the optical density, and *N* is the doping concentration of rare-earth ions. The theoretical oscillator strength equation is as follows:

(3) $$f_{cal}\left(J-J^{\prime }\right)=\frac{8\pi^{2}mc}{3h(2j+1)\lambda }\cdot \chi_{ed}\cdot S_{ed}=\frac{8\pi^{2}mc}{3h(2J+1)\lambda }\cdot \frac{{\left(n^{2}+2\right)}^{2}}{9n}\sum_{t=2,4,6}{\Omega_{t}\left|\langle SLJ\left\|U_{\left(t\right)}\right\|S^{\prime }L^{\prime }J^{\prime }\rangle \right|}^{2}$$

where *|<SLJ||U_(t)_|||S′L′J′>|* is the electric dipole transitions approximation matrix, and *U*_(*t*)_ is the transition matrix element for rare-earth ions, independent of the matrix composition of the experimental samples. The transition matrix elements corresponding to Er^3+^ ions are shown in Table 2. $\chi_{ed}$ is the correction factor for the electric dipole radiative transitions, *S_ed_* is the line intensity of the electric dipole, and Ω*_t_* is the J-O intensity parameter.

Assuming $f_{exp}=f_{cal}$, one can obtain multiple sets of J-O intensity parameters Ω*_t_* (*t* = 2, 4, 6). The determined value of Ω*_t_* can be obtained by least squares fitting.

Let the scalar function be:

(4) $$Y=\sum_{i=1}^{6}{\left\{S_{ed}\left(i\right)-\left[\Omega_{2}U\left(i,1\right)+\Omega_{4}U\left(i,2\right)+\Omega_{6}U\left(i,3\right)\right]\right\}}^{2}$$

where Ω*_t_* (*t* = 2, 4, 6) is required to minimize the scalar function, and according to the method of least squares, a system of equations is established:

(5) $$\frac{\partial Y}{\partial \Omega_{2}}=0,\frac{\partial Y}{\partial \Omega_{6}}=0,\frac{\partial Y}{\partial \Omega_{6}}=0$$

The Ω_2_ of the glass is calculated to be 2.92, Ω_4_ to be 1.44 and Ω_6_ to be 0.91, all in units of 10^−20^ cm^2^. Following the same methodology, the J-O strength parameters of the glass–ceramics heat-treated for different times are obtained as shown in Table 3:

Since the Ω*_t_* parameter is influenced by the ligand field environment surrounding the trivalent lanthanide ions, it is especially suitable for evaluating the local environment of trivalent rare-earth ions in glass [28]. As shown in Table 3, this parameter undergoes significant changes before and after processing. For glass–ceramics prepared at the same temperature, even with different heat treatment durations, the variations in the parameters are evident. With increasing heat treatment time, the value of Ω_2_ decreases, indicating a significant change in the microstructure around the trivalent Er^3+^ ions in the glass samples after heat treatment. This is because the covalency of the chemical bonds between trivalent lanthanide ions and their ligands strengthens as the Ω_2_ value increases [29]. Given that the electronegativity of F^−^ ions is higher than that of O^2−^ ions, the covalency of Er-O bonds is stronger than that of Er-F bonds [30]. However, with increased heat treatment time, the decrease in the Ω_2_ value suggests a weakening of the covalency of Er-O bonds and a relative strengthening of the covalency of Er-F bonds. This indicates that the Er^3+^ ions form crystal phases by bonding with F^−^ ions, signifying the migration of rare-earth ions from the glass phase to the crystal phase.

### 3.3. Fluorescence Spectroscopy

**Figure 4:**
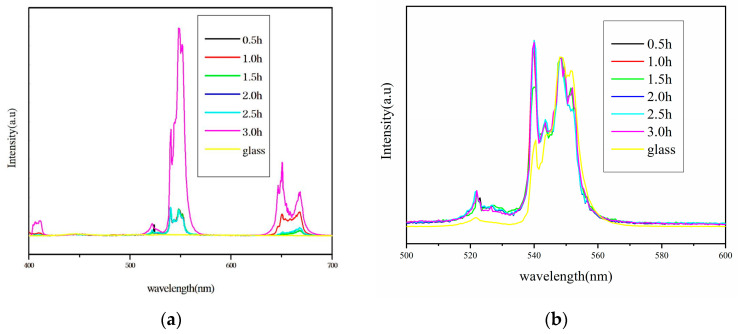
(**a**) Fluorescence spectra of Er^3+^-doped glass–ceramics. (**b**) Fluorescence spectrum after normalization at 545 nm.

Figure 4a shows the fluorescence spectra of Er^3+^-doped glass and glass–ceramic samples in the wavelength range of 400 nm to 700 nm. When excited by a 980 nm laser, the emission intensity of the glass sample is very low. However, when the glass is converted into a glass–ceramics through heat treatment, the emission intensity significantly increases. The spectrum displays four sharp peaks located at 410 nm, 520 nm, 545 nm, and 652 nm. Although the positions of the emission peaks remain unchanged, their intensity increases with the extension of heat treatment time, reaching a maximum at 3 h. This indicates that the luminescence of the glass–ceramics mainly originates from the Er^3+^ ions in the crystal phase, suggesting that the amount of rare-earth ions migrating from the glass phase to the crystal phase increases with prolonged heat treatment time.

Figure 4b presents the normalized fluorescence spectra at 545 nm, aimed at illustrating the presence of Er^3+^ ions in the crystal phase after heat treatment [31,32,33,34]. By normalizing the fluorescence spectra, it becomes possible to make clearer comparisons of spectral characteristics among different samples. The normalized spectral intensity of the glass sample is lower than others, a phenomenon associated with Stark level splitting. Analyzing the overall trend, from the relationship between fluorescence intensity (***I***) and spontaneous emission transition probability (***A_JJ’_***):$I\propto A_{JJ^{\prime }}$, it can be seen that the integrated area in Figure 4b correlates positively with the changes in the J-O intensity parameters, making quantitative analysis of the intensity parameters feasible.

Based on the calculated J-O intensity parameter Ω*_t_*, the spontaneous radiative excursion chances between the *J-J’* energy levels can be calculated as follows:

(6) $$A_{JJ^{\prime }}=A_{ed}=\frac{64\pi^{4}e^{2}}{3h(2J+1)\lambda^{3}}\cdot n^{2}\chi_{ed}S_{ed}$$

According to statistical physics, the ratio of the number of particles in the ^2^H_11/2_ and ^4^S_3/2_ energy levels of which Er^3+^ conforms to Boltzmann statistics is given. For the system of nearly independent particles, the number of particles at different energy levels conforms to the Boltzmann distribution:

(7) $$N_{l}=\omega_{l}e^{-\alpha -\beta }$$

The fluorescence intensity of the energy level leap is denoted by *I*:

(8) $$I=A_{JJ^{\prime }}\cdot N_{l}$$

Define *Ratio_obs_* as the fluorescence intensity ratio of 520 nm and 545 nm green luminescence produced by the upconversion process:

(9) $${Ratio}_{obs}=\frac{I_{1}}{I_{2}}$$

Associating the above equations and combining them with the J-O theory yields:

(10) $$Ratio=\frac{0.2225\Omega_{6}}{0.7158\Omega_{2}+0.4138\Omega_{4}+0.0927\Omega_{6}}\left(\frac{\lambda_{520\mathrm{nm}}}{\lambda_{545\mathrm{nm}}}\right)e^{-\frac{\Delta E}{kT}}\;=\frac{0.2225\Omega_{6}}{0.7158\Omega_{2}+0.4138\Omega_{4}+0.0927\Omega_{6}}\times 33.221$$

The temperature (*T*) is taken as room temperature. The excitation wavelength is 980 nm. The power is 0.5 W, and *k* is the Boltzmann constant.

From the fluorescence spectra, it is observed that the emission intensity of the glass is very low. After normalizing the fluorescence spectra, it was found that the peak area ratio at 520 nm and 545 nm is the same for both the glass and glass–ceramic samples; however, the fluorescence intensity of the glass sample is only one-thirtieth of the glass–ceramic sample. This is because the phonon energy of PbF_2_ crystals is much lower than the glass matrix, reducing the non-radiative relaxation rate of Er^3+^ ions in PbF_2_ crystals and enhancing the upconversion luminescence performance. Regardless of whether the rare-earth ions are located in the glass phase or the crystal phase, they can be detected through absorption spectra. However, the J-O parameters calculated from the absorption spectra are mixed, incorporating information from both the glass and crystal phases. Thus, when calculating the fluorescence intensity ratio of the green light emissions at 520 nm and 545 nm produced by upconversion processes, the result obtained using J-O parameters reflects the fluorescence intensity ratio of the entire sample, whereas the ratio derived from the peak areas in the fluorescence spectra represents only the fluorescence intensity ratio of the crystal phase.

Define *Ratio_lum_* as the theoretical value, which is the ratio calculated from the peak areas at 520 nm and 545 nm in the fluorescence spectra; and *Ratio_obv_* as the experimental value, which is obtained by substituting the J-O intensity parameters into Equation (10). According to the data in Table 4, between heat treatment durations of 0.5 h to 2.5 h, there is a significant difference between the theoretical and experimental values, indicating that the rare-earth ions are not only present in the crystal phase but also partly in the glass phase. As the heat treatment time increases, the rare-earth ions gradually migrate into the crystal phase, thereby reducing their presence in the glass phase. At 3 h, the theoretical and experimental values are closest, suggesting that the process of rare-earth ions entering the crystal phase is largely complete at this point.

### 3.4. Comparison of the Glass Crystallization Process with the Entry of Er^3+^ into the Crystal Phase

In the glass-ceramic samples, some of the Er^3+^ ions are distributed in the glass phase, while others are in the crystal phase. The process of rare-earth ions entering the crystal phase is largely completed at 3 h, the number of Er^3+^ ions in the crystal phase reaches its maximum value. Let the content of Er^3+^ ions in the crystal phase at 3 h be 1, and the content of Er^3+^ ions that entered the crystal phase before 3 h be *X*, with the remaining ions in the glass phase as (1 − *X*). The J-O parameters for the glass are denoted as $\Omega_{2}^{\prime }$, and those for the glass-ceramic are $\Omega_{2}^{\prime \prime }$. The J-O parameter values obtained from the absorption spectra are substituted into Equation (11):

(11) $$X\cdot \Omega_{2}^{\prime \prime }+\left(1-X\right)\cdot \Omega_{2}^{\prime }=\Omega_{2}$$

The results of the calculations are shown in Table 5:

As shown in Table 5, the proportion of Er^3+^ ions in the crystal phase gradually increases with time. During the 0.5 h of heat treatment, the proportion of Er^3+^ ions in the crystal phase is 30.7% of the maximum, and by 3 h, this migration process is essentially complete. Compared to the glass crystallization process, the migration of rare-earth ions from the glass phase into the crystal phase is a slow process.

**Figure 5:**
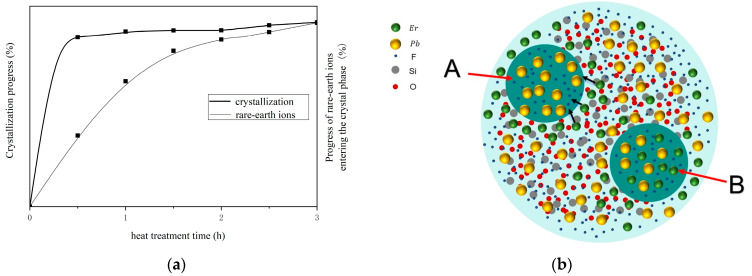
(**a**) Curves of the variation of crystallization progress and the progress of rare-earth ions entering the crystal phase with heat treatment time. (**b**) Schematic distribution of Er^3+^ ions within the SiO_2_-PbF_2_ glass system.

The crystallization process of glass–ceramics involves two stages: nucleation and growth. As shown in Figure 5a, when heat treatment is performed at the transformation temperature (T_g_), the glass sample initially forms nuclei rapidly, followed by the growth of these nuclei. As the nuclei continue to grow, the crystallinity of the sample increases. Once the nuclei reach their maximum size, the crystallinity of the sample will also approach its maximum. However, in glass–ceramics, rare-earth ions are not entirely distributed within the crystal phase; some remain in the glass phase. When the number of rare-earth ions in the crystal phase reaches saturation, the rare-earth ions in the glass phase will no longer continue to migrate into the crystal phase. As illustrated in Figure 5b, Area A represents the state where Er^3+^ ions are entering the crystal phase but have not yet reached their maximum quantity; Area B represents the state where the quantity of Er^3+^ ions in the crystal phase has reached its maximum, meaning no additional Er^3+^ ions will enter the crystal phase. Transitioning from State A to State B takes approximately 3 h, which is a relatively slow process compared to the crystallization of the glass. This indicates that the crystallization of the glass and the migration of rare-earth ions into the crystal phase do not occur synchronously through heat treatment.

## 4. Conclusions

In summary, we prepared glass-ceramic samples with a molar composition of 45SiO_2_-55PbF_2_-1Er_2_O_3_ using the high-temperature solid-state method. The differential scanning calorimetry (DSC) curve of the glass sample was analyzed to determine the optimal heat treatment temperature, which was found to be 460 °C. X-ray diffraction (XRD) analysis confirmed the presence of PbF_2_ crystals in the samples after heat treatment. Based on the electric dipole transition characteristics of Er^3+^ ions, the Judd–Ofelt theory was applied to calculate the Judd–Ofelt parameters for the Er^3+^ ions. Fluorescence spectra showed that the emission intensity of the untreated glass sample was low under 980 nm excitation, indicating that upconversion fluorescence primarily originates from Er^3+^ ions within the crystal phase. By combining the Judd–Ofelt parameters with the normalized peak areas and intensity ratios of the emissions, we found that after 3 h of heat treatment, the rare-earth ions were essentially fully incorporated into the crystal phase. The results indicated that the incorporation of Er^3+^ ions into the crystal phase was a relatively slow process. In contrast to the XRD analysis, which showed that the transformation from glass to glass–ceramics was rapid, the incorporation of rare-earth ions into the crystal phase occurred progressively. During the heat treatment, the glass sample rapidly nucleated and grew, while the rare-earth ions gradually entered the crystal phase, suggesting that these two processes did not occur simultaneously. After crystallization was complete, the heat treatment time significantly influenced the concentration of rare-earth ions in the crystal phase, thereby determining the luminescence intensity of the material. This study, using Er^3+^ ions as a model, explored their environment within glass–ceramics, providing a reference for the application of other rare-earth ions in similar materials and broadening the scope of rare-earth ion applications. Moreover, this work has significant theoretical value for optimizing the luminescent properties of rare-earth-doped glass-ceramic materials.

## Figures and Tables

**Table 1:** Diffraction peaks and glass peak areas.

	S	Diffraction Peak S_1_ (2θ = 26°)	Amorphous Halo S_2_	Crystallinity (%)
Time (h)				
0.5		87.1	294.7	29.6
1.0		159.4	520.5	30.6
1.5		152.1	493.2	30.8
2.0		178.6	579.7	30.8
2.5		198.7	626.6	31.7
3.0		189.0	585.9	32.2

**Table 2:** Er^3+^ transition matrix elements.

*U*_(*λ* = 2,4,6)_	^4^S_3/2_ → ^4^I_15/2_	^4^F_7/2_ → ^4^I_15/2_	^2^H_11/2_ → ^4^I_15/2_	^4^F_9/2_ → ^4^I_15/2_	^4^I_9/2_ → ^4^I_15/2_	^4^I_11/2_ → ^4^I_15/2_
*U*_(2)_	0	0	0.7158	0	0	0.0276
*U*_(4)_	0	0.1465	0.4138	0.5511	0.1587	0.0002
*U*_(6)_	0.2225	0.6272	0.0927	0.4621	0.0072	0.3924

**Table 3:** J-O strength parameters of Er^3+^-doped glass–ceramics.

Sample	Ω_2_ (10^−20^ cm^2^)	Ω_4_ (10^−20^ cm^2^)	Ω_6_ (10^−20^ cm^2^)
0.5 h	2.31	1.24	0.84
1.0 h	1.42	0.94	0.75
1.5 h	1.27	0.89	0.73
2.0 h	1.11	0.84	0.72
2.5 h	1.03	0.81	0.70
3.0 h	0.93	0.78	0.70

**Table 4:** Theoretical and experimental values.

	0.5 h	1.0 h	1.5 h	2.0 h	2.5 h	3.0 h
*Ratio_lum_*	4.98	5.02	5.01	4.96	4.99	5.02
*Ratio_obv_*	2.77	3.76	4.01	4.41	4.55	4.91

**Table 5:** Ratio of the number of Er^3+^ ions entered into the crystal phase to the maximum number of Er^3+^ ions in the crystal phase.

Time	0.5 h	1.0 h	1.5 h	2.0 h	2.5 h	3.0 h
*X*	30.7%	75.4%	82.9%	90.9%	94.9%	100%

## Data Availability

All data can be accessed upon request to the corresponding author.
